# Reduced gray matter volume and cortical thickness associated with traffic-related air pollution in a longitudinally studied pediatric cohort

**DOI:** 10.1371/journal.pone.0228092

**Published:** 2020-01-24

**Authors:** Travis Beckwith, Kim Cecil, Mekibib Altaye, Rachel Severs, Christopher Wolfe, Zana Percy, Thomas Maloney, Kimberly Yolton, Grace LeMasters, Kelly Brunst, Patrick Ryan

**Affiliations:** 1 Molecular Epidemiology in Children’s Environmental Health Training Program, Department of Environmental Health, University of Cincinnati College of Medicine, Cincinnati, Ohio, United States of America; 2 Imaging Research Center, Department of Radiology, Cincinnati Children’s Hospital Medical Center and the University of Cincinnati College of Medicine, Cincinnati, Ohio, United States of America; 3 Division of Biostatistics and Epidemiology, Department of Pediatrics, Cincinnati Children’s Hospital Medical Center and the University of Cincinnati College of Medicine, Cincinnati, Ohio, United States of America; 4 Department of Psychology, Western Kentucky University, Bowling Green, Kentucky, United States of America; 5 Department of Environmental Health, University of Cincinnati College of Medicine, Cincinnati, Ohio, United States of America; 6 Division of General and Community Pediatrics, Department of Pediatrics, Cincinnati Children’s Hospital Medical Center and the University of Cincinnati College of Medicine, Cincinnati, Ohio, United States of America; Nuovo Ospedale Prato (NOP) Santo Stefano, USL Toscana Centro, ITALY

## Abstract

Early life exposure to air pollution poses a significant risk to brain development from direct exposure to toxicants or via indirect mechanisms involving the circulatory, pulmonary or gastrointestinal systems. In children, exposure to traffic related air pollution has been associated with adverse effects on cognitive, behavioral and psychomotor development. We aimed to determine whether childhood exposure to traffic related air pollution is associated with regional differences in brain volume and cortical thickness among children enrolled in a longitudinal cohort study of traffic related air pollution and child health. We used magnetic resonance imaging to obtain anatomical brain images from a nested subset of 12 year old participants characterized with either high or low levels of traffic related air pollution exposure during their first year of life. We employed voxel-based morphometry to examine group differences in regional brain volume, and with separate analyses, changes in cortical thickness. Smaller regional gray matter volumes were determined in the left pre- and post-central gyri, the cerebellum, and inferior parietal lobe of participants in the high traffic related air pollution exposure group relative to participants with low exposure. Reduced cortical thickness was observed in participants with high exposure relative to those with low exposure, primarily in sensorimotor regions of the brain including the pre- and post-central gyri and the paracentral lobule, but also within the frontal and limbic regions. These results suggest that significant childhood exposure to traffic related air pollution is associated with structural alterations in brain.

## Introduction

Accumulating evidence suggests traffic-related air pollution (TRAP) is a contributor to both neurodegenerative diseases and neurodevelopmental disorders [1–9]. TRAP consists of a complex mixture of gaseous pollutants, fine and ultrafine particulate matter, heavy metals, elemental and organic carbon, polycyclic aromatic hydrocarbons, and other dynamic constituents [10]. Diesel exhaust (DE) is a significant contributor to TRAP with a composition incorporating ultrafine particulate matter (UFPM; <100 nm) and more than 40 toxic pollutants [4, 10]. UFPM can readily access the brain directly through the nasal olfactory mucosa via the olfactory bulb; this direct entry sets up a scenario for adverse structural consequences to occur in the brain due to the presence of TRAP despite a potentially low translocation rate from deposition in the nasal cavity [11–13].

Advancements in computational image analysis methods [14–16] reveal typical brain development as well as advance discovery of pathophysiological mechanisms associated with aberrant development and injury, including those from environmental exposures. Structural magnetic resonance imaging (MRI) derived cortical thickness assessment estimates the distance from the pial surface to the gray/white interface surface via automated reconstruction methods. Cortical thickness measures reflect the size, density and arrangement of neurons, neuroglia and nerve fibers while also reflecting axon and dendrite remodeling, and myelination as myelin proliferation into the cortical neutrophil replaces gray matter during development [17–21]. The measurement of cortical thickness [17, 22] pairs well with other whole brain MRI analysis techniques such as voxel based morphometry (VBM) which determines brain volume based on tissue class via a voxel-wise comparison of anatomical images [17–21]. VBM allows for detection of regional and global differences in volume including decreases or increases in gray and/or white matter [23–25]. For gray matter, VBM includes cortical surface area and cortical thickness. These complimentary methods characterize brain structure [25].

By comparing adolescents exposed to high and low concentrations of TRAP during the first year of life, we ascertained if there was evidence that early life exposure to TRAP was associated with changes in brain structure, specifically brain volume and cortical thickness. We hypothesized that participants with the highest early life TRAP exposures would demonstrate atypical neural development with reduced regional brain volumes and cortical thickness at age 12 years compared to those with lowest TRAP exposures. If early life TRAP exposure irreversibly harms brain development, as with infection or teratogen exposure, structural consequences could persist regardless of the time point for a subsequent examination. However, the study was exploratory as identification of regions with alterations in brain volume and cortical thickness informs potential mechanisms and provides functional significance on how pollutants exert their effects in the brain.

## Materials and methods

### Study enrollment

Participants in this study are a subset of the previously described Cincinnati Childhood Allergy and Air Pollution Study (CCAAPS) cohort [26, 27]. Briefly, CCAAPS is a prospective cohort study of children recruited prior to age 6 months to examine early childhood exposure to TRAP and health outcomes including allergic diseases, asthma, and neurodevelopment. Eligibility for study enrollment included participants born at a gestational age greater than 35 weeks, a birth record address either < 400 meters (m) or > 1500 m from a major highway, and at least one biological parent with an allergic disease. Participating children and their caregivers completed clinical visits at ages 1, 2, 3, 4, 7, and 12 years (y). Caregivers completed study questionnaires at each study visit regarding their child’s health and general wellbeing, housing characteristics, and residential history. At all study visits children completed allergy testing and physical examinations, including assessment for growth, anthropometry, and developmental milestones. The clinic visit at age 12 y included direct and indirect assessments of intelligence, reading ability, executive function, mental health, and other neurodevelopmental outcomes. A nested imaging substudy was conducted on subset of CCAAPS participants at the age 12 y study visit with eligibility details described below. The Institutional Review Boards at the University of Cincinnati and the Cincinnati Children’s Hospital Medical Center (CCHMC) approved the study protocol. Participants provided written assent prior to participation. Participant’s parents and legal guardians provided informed consent prior to participation.

### Exposure to traffic-related air pollution

Childhood exposure to TRAP was estimated for study participants using a previously developed and validated land-use regression (LUR) model [28, 29]. Briefly, an ambient air sampling network consisting of 27 sampling sites was operated from 2001–2006 in the greater Cincinnati area, and 24-hour sampling was conducted simultaneously at 4–5 sites over different seasons [29]. Particulate matter less than 2.5 micrometers (PM_2.5_) samples were collected on 37-mm Teflon membrane filters and 37-mm quartz filters with Harvard-type Impactors. PM_2.5_ mass concentrations were determined by gravimetric analysis [30]. Teflon filters were analyzed by X-ray fluorescence to determine the elemental concentration of 39 elements and quartz filters were analyzed by thermal-optical transmittance using the NIOSH-5040 method to determine elemental and organic carbon concentrations. A multivariate receptor model, UNMIX, was used to identify significant sources contributing to PM_2.5_ concentrations, including traffic. In order to estimate the specific fraction of the sampled elemental carbon arising from diesel exhaust, we applied elemental source profiles obtained from measurements conducted at cluster sources of diesel-fueled trucks and buses [30]. Thus, the fraction of the sampled elemental carbon due to diesel combustion was derived from each sampling day and is referred to as the elemental carbon attributable to traffic (ECAT), [30, 31]. Daily ECAT concentrations averaged over the entire monitoring period at the sampling sites ranged from 0.22–1.02 μg/m^3^ and served as the dependent variable in our LUR model development [29]. LUR predictor variables included elevation, nearby truck traffic, and bus routes [29]. The final LUR model was applied to all residential locations of CCAAPS participants beginning at the birth record address and through age 12 y as reported by caregivers to derive time-weighted estimates of ECAT exposure throughout childhood [28, 29].

### Nested imaging substudy eligibility

High-resolution anatomical imaging was acquired on a subset of CCAAPS participants who completed the age 12 y study visit. The intent of the nested imaging substudy was to identify differential imaging outcomes in children exposed to high levels of TRAP during early childhood compared to children exposed to low TRAP levels. Therefore, eligible participants whose estimated ECAT exposure from birth through age one year were in the highest or lowest quartiles of exposure, or whose birth record address was less than 400 m from a major road were recruited to participate in the nested imaging substudy. These participants were contacted about completing the imaging when scheduling the 12 y clinic visit, screened for exclusions or contraindications to MRI, such as non-removable dental appliances, braces, implanted devices, or known to be claustrophobic, and enrolled in the nested imaging substudy.

### Participant characteristics

A total of 147 children were enrolled in the nested imaging substudy. Of these, imaging data was not available for 12 participants due to issues with image reconstruction, artifacts or incidental structural brain abnormalities that interfere with image processing. Demographic and other characteristics of the 135 participants included in this analysis are presented in Table 1. Overall, participants in the substudy were 56.3% male, 74.8% Caucasian, and were similar to participants who completed the age 12 clinic visit and the overall CCAAPS cohort with respect to demographic characteristics (Table 1). The majority were singleton births (127 (94%)) and right handed (126 (93%)). Table 2 presents the distribution of participant characteristics by early childhood TRAP exposure status. As expected from the design, the mean estimated ECAT exposure at the birth record address was twice as high in the high exposed group compared to the low exposed group (0.56 [0.13] versus 0.27 [0.02] μg/m^3^, respectively). In addition, a greater proportion of participants in the substudy with high ECAT exposure were more likely to be African-american with reported annual household incomes < $20,000 at initial study enrollment (Table 1).

**Table 1:** Characteristics of CCAAPS participants at enrollment, age 12 y, and imaging subset [n(%) or mean (SD)].

Characteristic (unit)	Enrollment	Age 12 y	MRI
	n = 762	n = 344	n = 135
*Child characteristics*	* *		
Sex			
Male	415 (54.5%)	191 (55.5%)	76 (56.3%)
Female	347 (45.5%)	153 (44.5%)	59 (43.7%)
Race / Ethnicity			
Caucasian	587 (77.0%) (77.4%)	261 (75.9%)	101 (74.8%)
African American / More than one race	175 (23.0%) (22.6%)	83 (24.1%)	34 (25.2%)
Birth weight (lbs)	7.5 (1.2)	7.6 (1.2)	7.6 (1.2)
Duration of breastfeeding (months)	5.7 (6.5)	6.3 (6.9)	5.6 (6.9)
*Maternal characteristics*	* *		
Age at study enrollment (years)	30.0 (5.7)	30.7 (5.9)	29.7 (5.7)
Maternal education at child age 1			
≤ High school	185 (24.9%)	72 (21.6%)	36 (27.7%)
Some college or trade school	196 (26.4%)	94 (28.1%)	31 (23.9%)
≥ College degree	361 (48.7%)	168 (50.3%)	63 (48.5%)
*Household characteristics*	* *		
Household income (Parental report at first study visit)*			
< $20,000	129 (17.5%)	58 (17.5%)	28 (21.5%)
$20,000 to < $40,000	129 (17.5%)	54 (16.3%)	22 (16.9%)
$40,000 to < $90,000	210 (28.5%)	95 (28.6%)	33 (25.4%)
$90,000 to < $110,000	196 (26.6%)	89 (26.8%)	36 (27.7%)
> $110,000	73 (9.9%)	36 (10.8%)	11 (8.5%)
ECAT at birth record address (μg / m^3^)	0.39 (0.13)	0.39 (0.14)	0.44 (0.18)

*Missing values (~3%) were not reported by parents

**Table 2:** Model variables evaluated in the nested imaging substudy cohort.

Characteristic	Cohort (N = 135)	Range	Low exposure (N = 59)	High exposure (N = 76)	F or X^2^	P-value
Race: African American^1^	34 (~25%)		6 (~10%)	28 (~37%)	12.54	0.0004
Sex^1^	59 Female (~44%)		29 Female (~49%)	30 Female (~39.5%)	1.26	0.2608
ECAT at birth record address^2^	0.44 (±0.17)	0.24–0.88	0.27 (±0.02)	0.56 (±0.13)	286.06	< 0.0001
ECAT at imaging^2^	0.38 (±0.13)	0.24–0.83	0.30 (±0.06)	0.44 (±0.14)	54.20	< 0.0001
Average ECAT^2^	0.39 (±0.13)	0.24–0.85	0.29 (±0.03)	0.48 (0.11)	163.33	< 0.0001
Child birth weight (pounds)^2^	7.62 (±1.26)	4.44–10.90	7.86 (±1.31)	7.42 (±1.19)	4.20	0.0423
Child age at imaging (years)^2^	12.12 (±0.75)	11.0–14.71	12.23 (±0.80)	12.03 (±0.7)	2.43	0.1211
Maternal age (years)^2^	29.7 (±5.7)	18.34–43.1	31.95 (±5.01)	27.96 (±5.62)	18.44	< 0.0001
Child FSIQ^2^	99.32 (±15.9)	44–139	101.9 (±15.33)	97.3 (±16.13)	3.91	0.097
Maternal IQ^2^	105.11 (±13.28)	65–145	109.24 (±12.02)	101.91 (±13.39)	10.87	0.0013
Deprivation index at birth^2^	0.45 (±0.19)	0.18–1.0	0.36 (±0.1)	0.53 (±0.21)	29.80	< 0.0001

Data presented as mean (± S.D.) or n (%)

F, Χ^2^, and P-values represent comparison between low and high exposure groups

^1^ Chi-square test

^2^ One-way ANOVA

### Image acquisition

The participant MRI examinations were acquired using a 3T Achieva scanner (Philips Medical Systems, Best, Netherlands) equipped with a 32-channel head coil. High-resolution, three dimensional, anatomical imaging data were collected using a single-shot turbo field echo (TFE) pulse sequence operating with an 8.2 milliseconds (ms) repetition time, a 3.7 ms echo time, a 1057 ms inversion time, a 8º flip angle, a sensitivity encoding (SENSE) factor of 2 (right-left) and 1 mm^3^ resolution.

### Image processing

Anatomical images were reconstructed using Cincinnati Children’s Image Processing Software (https://irc.cchmc.org/software/cchips.php) running in IDL 8.1 (Exelis Visual Information Solutions, Boulder CO). Images were then imported into Statistical Parametric Mapping 12 (SPM12) version 6685 (Wellcome Department of Cognitive Neurology, London) running in Matlab 7.13.0.564 (The Mathworks, Inc., Natick, MA). All images were first visually scanned for artifacts and any other abnormalities. Images were then manually reoriented so that the anterior and posterior commissures were in the same axial slice with the origin voxel [0, 0, 0] set medially on the anterior commissure. This step ensures that all images are in the same general orientation prior to processing and reduces the number of errors encountered during processing. Reoriented images were then processed using the Computational Anatomy Toolbox (CAT) version r1113 (http://www.neuro.uni-jena.de/cat/) for SPM12 [32]. A more detailed description of the initial image processing can be found in the methods of Beckwith, Dietrich [33]. Please see S1 Fig. for a simplified visual of the CAT12 workflow. Briefly, images were skull stripped, reoriented to a template in Montreal Neurological Institute (MNI) space using affine registration, segmented into tissue classes, and processed to minimize noise and partial volume effects [34]. The diffeomorphic anatomical registration through exponentiated line algebra (DARTEL) toolbox [35] within SPM12 was then used to apply a nonlinear deformation to the images utilizing an IXI-database template (http://www.brain-development.org). Normalized images were then bias-corrected and modulated by scaling the Jacobian determinants to account for differences in the tissue volume that occur during normalization. Normalized gray and white matter tissue probability maps were smoothed using an 8 mm Gaussian kernel.

The calculation of cortical thickness is included as an optional step in the VBM segmentation pipeline for the CAT toolbox. A projection based thickness [36] technique estimates the white matter distance during the segmentation procedure and projects the local maxima onto neighboring gray matter voxels using the relationship to that distance. This distance is representative of cortical thickness. This technique permits the use of partial volume data, as well as sulcal asymmetries and blurring without requiring sulcal reconstruction [37]. To account for topological variations, a technique utilizing spherical harmonics was incorporated [38]. Spherical mapping [39] was used to permit the use of a shared coordinate system, and an adaptation of the DARTEL algorithm [35] was used for surface registration [40]. Cortical thickness images were then smoothed to 15 mm as recommended in the CAT12 manual.

### Statistical analyses

Demographic characteristics, imputations of missing demographic variables, collinear relationships and covariate selection for the models were carried out in SAS software (SAS Institute, Cary, NC). Comparisons between participant characteristics were made using a Chi-Square test for categorical variables, and a one-way analysis of variance (ANOVA) for all other variables. Missing data points were generated using a multivariate normal distribution multiple imputation model in SAS. The rate of missing variables was not predicted by any variables in the imputation analysis and data points were assumed to be missing at random. Ten data sets were imputed using the PROC MI procedure. Parameter estimates for each imputed data set were generated via a general linear model and a pooled analysis was conducted to estimate the standard error for the imputed data set and compared to the initial estimate.

To account for the spatial features within the imaging analyses, we also employed SPM12 running in Matlab for the statistical analyses relating to imaging derived cortical thickness and VBM. Within SPM12, a full-factorial model allowed for differences in cortical thickness, gray matter volume, and white matter volume between the two exposure groups. Participants were grouped by exposure status (low ECAT, high ECAT) and sex. Main effects for exposure status and sex were initially explored using an omnibus F-test. An F-test was also used to explore the possibility of an interaction between exposure status and sex. In the event of a statistically significant finding, post-hoc T-tests were used to establish the directionality of the effect. Threshold-Free Cluster Enhancement (TFCE) [41, 42] was utilized for cluster-based statistics. Thresholds for the analyses were set using a Family-Wise Error (FWE) corrected P-value of 0.05 to correct for multiple comparisons.

### Covariate selection

Variables considered for inclusion as covariates were: age at time of MRI examination, handedness, gestational age, birth weight, maternal IQ ascertained using the Wechsler Abbreviated Scale of Intelligence– 2^nd^ Edition, participant full scale IQ obtained using the Wechsler Intelligence Scale for Children–Fourth Edition (WISC-IV), race, sex and a previously calculated index measure of census tract deprivation index at birth [43]. Variables at the census-tract level contributing to the deprivation index include the median household income, fraction of households with income below the poverty level, educational attainment, health insurance coverage, households receiving public assistance and vacant houses. Sex was included as a factor in the analyses. Age at MRI examination [44], birth weight [45, 46], participant full scale IQ [47], census tract deprivation status at birth [43], and race [48, 49] were selected for inclusion in the final model. Total intracranial volume was also included as a covariate for volumetric analyses. Furthermore, because aspects of socioeconomic status were incorporated into the census tract deprivation index metric, variables such as income and educational attainment were not included as separate covariates in the final analyses.

### Data and code availability statement

The data and code used in the study are not available in the public domain. Data usage is currently governed by the Institutional Review Board at Cincinnati Children’s Hospital Medical Center. The data contains sensitive information and is confidential. The investigators will share de-identified data following approved institutional review board (IRB) policies. Investigators may request de-identified data by contacting the corresponding author. The CCHMC IRB can be reached at irb@cchmc.org, ORCRA@cchmc.org, or by phone at 513.636.8039.

## Results

**Fig 1:**
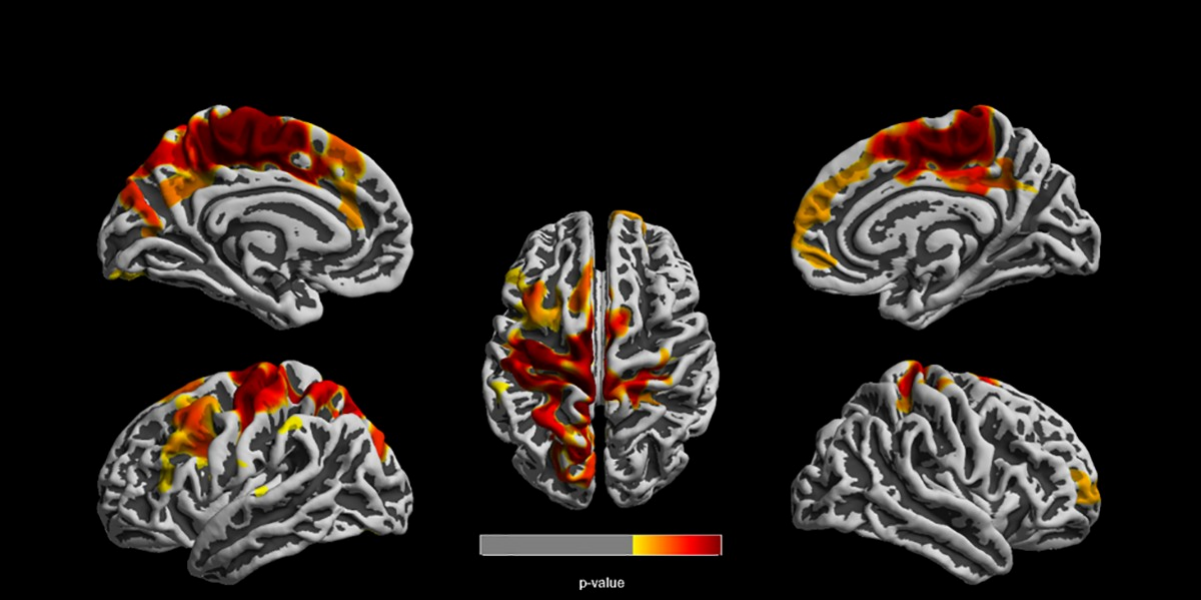
Statistically significant clusters using threshold free cluster enhancement. Clusters represent reduced cortical thickness in the high ECAT group compared to the low ECAT group. Clusters were corrected for multiple comparisons using a familywise error rate of p ≤ 0.05.

Upon direct comparison, we found decreased cortical thickness in the high ECAT group relative to the low ECAT group before and after adjusting for covariates. The reduction in cortical thickness for the somatosensory region was approximately 3% upon comparing the average values for high ECAT group relative to the low ECAT group. In the left hemisphere, regions of reduced cortical thickness were observed in the paracentral lobule, pre- and post-central gyri, precuneus, superior frontal gyrus, and superior parietal lobule (Fig 1, Table 3). In the right hemisphere, reduced cortical thickness was observed in the postcentral gyrus, paracentral lobule, posterior cingulate, superior frontal gyrus, and superior parietal lobule (Fig 1, Table 3). No main effects for sex were observed, nor was an interaction between ECAT exposure and sex present.

**Table 3:** Reduced cortical thickness in high ECAT exposure group compared to low ECAT exposure group with covariates.

**Anatomical region**	**Cluster size (vertices)**	**Peak vertex MNI coordinates (X, Y, Z)**	**Vertex p-value**
Left hemisphere			
Anterior cingulate Medial frontal gyrus Paracentral lobule Postcentral gyrus Precentral gyrus Precuneus Superior frontal gyrus Superior parietal lobule	31070	-16–43 56 -8–39 55 -4–17 59	0.006 0.006 0.006
Middle frontal gyrus Precentral gyrus	5350	-44 16 47 -34 10 29 -41 12 39	0.022 0.022 0.022
Fusiform gyrus	604	-21–92–12 -37–80–16	0.044 0.048
Inferior parietal lobule	279	8–33 4	0.049
Right hemisphere			
Cingulate Medial frontal gyrus Paracentral lobule Postcentral gyrus Superior frontal gyrus Ventromedial prefrontal cortex	16292	5–18 56 6–27 59 4–35 55	0.007 0.007 0.009
**Postcentral gyrus**	**191**	**44–19 38**	**0.049**

Vertices and clusters corrected for multiple comparisons (FWE) at P < 0.05 using TFCE; ECAT = Elemental carbon attributable to traffic; FWE = Familywise error rate; MNI = Montreal neurological institute; TFCE = Threshold free cluster enhancement

**Fig 2:**
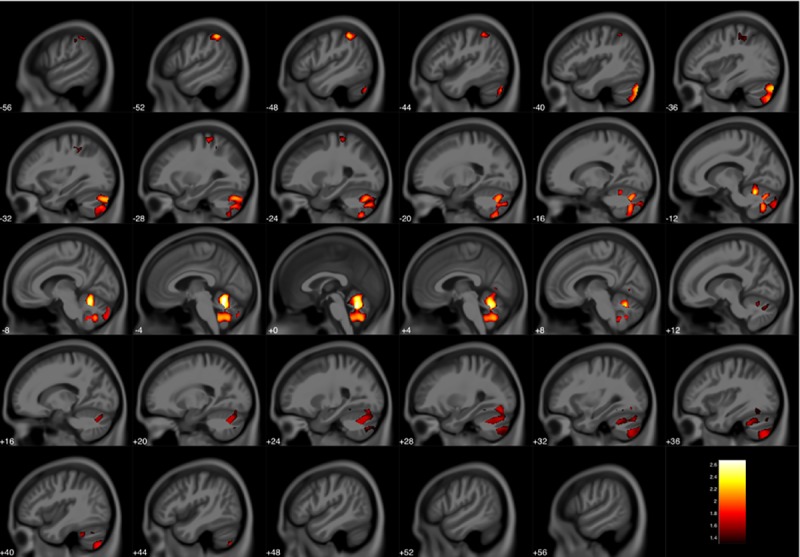
Reduced gray matter volume in the high ECAT group compared to the low ECAT group. Clusters were corrected for multiple comparisons using threshold free cluster enhancement with a familywise error rate of p ≤ 0.05. Color bar represents–log(p) value.

Volumetric analyses revealed reduced gray matter in the high versus low ECAT group before and after adjusting for covariates. Volumetric differences were observed in two regional clusters. The first cluster was located bilaterally in the cerebellum and extended to the right parietal cortex (Fig 2, Table 4). The second cluster was located primarily in the parietal lobe, including the pre- and post-central gyri, inferior parietal lobule, and supramarginal gyrus (Fig 2, Table 4). The volumetric reduction was approximately 4% upon comparing the average values for high versus low ECAT group. A small main effect for sex was also observed, with females displaying reduced gray matter volumes in frontal sub-lobar regions (not shown). However, no interaction between ECAT group and sex was detected. Furthermore, no volumetric differences were seen in any of the white matter analyses.

**Table 4:** Reduced gray matter volume in high ECAT exposure group compared to low ECAT exposure group with covariates.

**Anatomical region**	**Cluster size (voxels)**	**Peak voxel MNI coordinates(X, Y, Z)**	**Voxel p-value**
Left cerebellum	10731	-6–57–18 -2–56–26 -38–82–34	0.002 0.002 0.006
Right cerebellum	1258	40–70–54 32–70–46 30–84–44	0.016 0.020 0.028
	145	36–60–15 30–50–14	0.044 0.047
Inferior parietal lobule	711	-51–46 51 -56–33 44 -52–33 52	0.006 0.039 0.046
Precentral gyrus	226	-34–39 48 -34–30 50 -28–44 51	0.034 0.036 0.037
	251	-27–33 63	0.016

Voxel and clusters corrected for multiple comparisons (FWE) at P ≤ 0.05 using TFCE; ECAT = Elemental carbon attributable to traffic; FWE = Familywise error rate; MNI = Montreal neurological institute; TFCE = Threshold free cluster enhancement

## Discussion

### Overall findings

Our study found a bilateral, medial region of reduced cortical thickness within the posterior frontal and anterior parietal lobes with early life high exposure to TRAP. Within the posterior frontal lobe, the precentral gyrus serves as the primary motor cortex and is responsible for executing voluntary movements through connections to the spinothalamic tract [50]. Immediately posterior, the parietal lobe with the postcentral gyrus is the primary sensory area. It is a topographically organized, functionally defined area responsible for integrating somatosensory information such as touch and proprioception [51].

We also observed reduced gray matter volume in relation to increased TRAP exposure, primarily in the cerebellum, but also including the cerebellar vermis and the pre- and post-central gyri. This decrease was selective to gray matter and without a corresponding increase in white matter volume, which suggests the cerebellar changes are not due to a process of maturation, which would alter both gray and white matter. The cerebellum is primarily a modulatory brain region involved in the regulation of motor function, cognition, and emotion [52–56]. The cerebellum in particular grows rapidly in the first two years of life [57], with cerebellar injuries being related to cognitive developmental disorders [58] such as autism [59].

This combination of reduced cortical thickness primarily within the precentral gyrus and the reduced cerebellar volume implicates that the effects of TRAP impact two regions involved in motor function. Given these systems are early developing, they are more likely to be impacted by adverse insults such as environmental toxicants during critical developmental periods [60, 61]. Motor planning has been suggested to be related to cognitive performance and disorders such as anxiety [62–64].

### Supporting evidence of prenatal exposure to TRAP

Early life exposure to TRAP have been previously described. Transplacental exposure to air pollutants with DNA adducts of polycyclic aromatic hydrocarbons (PAH) were measured in umbilical cord blood [65–67]. Placental expression of brain-derived neurotrophic factor (BDNF) and synapsin 1 (SYN1), two genes implicated in normal neurodevelopmental trajectories, decreased with increasing in utero exposure to PM_2.5_ [68]. Direct or indirect maternal effects from air pollution, such as systemic low-grade inflammation, increased plasma viscosity, hormonal disruption or epigenetic changes, potentially impair placenta function and lead to neurological disruption by time of birth via mechanisms from decreased oxygen and nutrient transport [69].

### Evidence of postnatal TRAP within the human central nervous system

Besides direct inhalation in postnatal life, other possible mechanisms for TRAP to cause detrimental central nervous system effects include translocation from the pulmonary, gastrointestinal and circulatory systems [12, 70, 71]. Alterations in the blood brain barrier (BBB), disruption of endothelium, and microglial activation, accompanied by neuroinflammation, as well as the ability of TRAP to exert effects on the brain secondarily with cardiovascular dysfunction, all could potentially produce brain pathology [72–75]. Brain tissue from individuals, ages 2–45 years, living in highly polluted areas showed an increase in CD68, CD163, and HLA-DR antigens implicating infiltrating monocytes or resident microglia activation [75]. Upregulation of pro-inflammatory markers such as COX2 and IL1-β, and the CD14 markers for innate immune cells presented in the frontal cortex, vagus nerves and substantia nigra [75]. Increased Aβ42 deposition, BBB disruption, endothelial cell activation [75], and brain lesions in the white matter of the prefrontal lobe were observed [75, 76]. Adolescent brains also featured significant amounts of lipfuscin in endothelial cortical capillaries [75]. Further studies demonstrated elevations hyper-phosphorylated tau and α-synuclein [77].

### Evidence of TRAP exposure interfering with brain development in model systems

Early life TRAP exposures pose a substantial risk for interfering with normal brain development as constituent exposures may permanently harm the maturing cortex. Ejaz, Anwar [78] observed with histopathology and immunohistochemistry a positive dose-response relationship between PM exposure and severity of neuronal loss, predominately in the motor cortex and primary somatosensory cortex in rat models. Allen, Oberdorster [79] studied mice with an exposure models employing concentrated ambient ultrafine (UFP) particles with two exposure periods: postnatal day (PND) 4–7 and 10–13 (human 3rd trimester equivalent). This model employed the most reactive constituents measured in air pollution at levels representative of high traffic areas in major U.S. cities [79]. UFP exposures provoked inflammation and microglial activation, specifically, increased astrocytic activation in the amygdala [79]. Reductions in size of the corpus callosum were accompanied by hypomyelination, ventriculomegaly [79]. These models revealed elevated glutamate in both sexes, however, only males showed an altered ratio of glutamate and GABA with excitatory-inhibitory imbalance [79]. Finally, male mice in the models featured repetitive and impulsive behaviors [79]. The findings indicated the human 3rd trimester equivalent as potential susceptible to neurodevelopmental toxicity from UFP [79]. The results also supported the notion that exposure to UFP air pollution throughout periods of rapid neurodevelopment may increase the risk for developing ASD, and potentially other disorders such as ADHD, periventricular leukomalacia and schizophrenia [79].

### Epidemiology of air pollution effects on mental health with imaging features

In children, epidemiologic evidence also supports a link between air pollution and ASD, ADHD, schizophrenia, developmental and cognitive delays [80–83]. Our group found evidence that each 0.25 mg/m^3^ increase in early life TRAP was associated with increases in depression and anxiety scores for the CCAAPS cohort [84]. In adults, there is similar evidence associating air pollution with measures of anxiety and depression [85–87]. Gestational and early childhood exposure to TRAP is associated with higher risk for schizophrenia, low birth weight and ASD [88]. Individuals with childhood onset schizophrenia demonstrated diffuse decreases in mean cortical thickness, with deficits localizing more anteriorly [89]. As reported by Newman, Ryan [80] using the Behavior Assessment System for Children, 2^nd^ edition, exposure to the highest tertile of ECAT during the child’s first year of life was significantly associated with hyperactivity T-scores in the “at-risk” range at 7 years of age for those participants whose mothers had more than a high school education. ADHD has been associated with reductions in cortical thickness in prefrontal and precentral regions, and in the parietal lobe [90, 91]. The superior parietal lobule in particular appears to be heavily involved in attention [92–94] and is strongly associated with differences functional connectivity in ADHD [92, 95–98]. This may be due to a delay in the maturation [99] and functional development [97] of the cortex. Similarly, pediatric anxiety is related to differences in structural gray matter volumes [100], and Brunst, Ryan [101] found that myo-inositol may mediate anxiety levels in relation to TRAP exposure. Cerebellar abnormalities are consistently associated with numerous mental health disorders including anxiety, ADHD, ASD, and schizophrenia [55, 102–105]. Cerebellar volumes have been inversely correlated with depression and anxiety [106], and aberrant connectivity with other neural systems have been implicated as well [107, 108].

### Cortical development, timing, thickness and volume

Cortical brain development in humans relies upon the division of progenitor cells within the ventricular zone of the germinal matrix [109]. The derived neurons and glia cells migrate outward toward the cortical plate and undergo a series of morphological changes where the cells differentiate and integrate into functional circuits. Cortical neurogenesis and migration are completed by the first week of postnatal life [110–112]. Subsequently, cortical development is largely dependent on dendritic growth, growth of the terminal axon arborization, myelination, and synaptogenesis [113–118]. Cerebral lamination in the developing fetus and at birth relies on the appearance and the resolution of the subplate, with subplate neurons that serve as a crucial regulator of cortical development [119]. Insults damaging one or more underlying cellular events during neurogenesis and migration can produce a variety of cortical changes [120]. Cortical thinning itself may indicate a loss of dendrites and dendritic spines and changes in myelination within specific brain systems [121, 122]. However, changes in cortical thickness and brain volume appropriately occur from in utero into adulthood with distinct regional timetables revealed noninvasively with fetal and postnatal neuroimaging [123]. During childhood, volumetric changes can occur from changes in neuronal size, neuropil, dendritic or axonal arborization or from the vasculature, synaptic proliferation and pruning, along with increasing myelination [124]. In the first two years of life, the human cerebral cortex undergoes marked expansion with the cortical surface area essentially doubling between birth and age 2 [118]. Lyall, Shi [125] found that cortical thickness by age 2 years reaches an estimated 97% of adult values, yet the corresponding cortical surface area is estimated at 69% adult values. Amlien, Fjell [126] explained cortical expansion of surface area and thickness across primate species as adhering to general allometric laws of scaling. They observed that cortical thickness showed a continuously negative trajectory for the range of 4 to 30 years of age across the entire cortex. In contrast, cortical surface area was positively related to age until about 12 years, with little subsequent differences. Gogtay, Giedd [127] conducted anatomical MRI examinations every two years between the ages of 4 and 21 years for 13 participants. Maturation of the cortex followed the evolutionary sequence in which the regions were created. Gray matter volumes peaked the earliest in primary sensorimotor areas and the latest in higher order association areas. The participants in the current study were imaged at age 12 years, which further supports that our findings are not related to changes occurring with the completion of typical cortical maturation associated with age, especially for sensorimotor regions.

### Limitations

While our cohort is part of an ongoing, longitudinal evaluation of TRAP, our structural outcomes were ascertained based upon only one MRI examination and may be influenced by interindividual variance or cohort effects. The high-low design targeting exposure during a given period (first year of life) and time of imaging at the same age (12 y) attempted to minimize the effects of changes in developmental maturation and unaccounted individual variances. However, if both groups are adversely impacted by TRAP, our approach may underestimate the effects due to our design. The regional specificity of the pre- and post-central gyri and the cerebellar gray matter findings with the absence of white matter volumetric findings suggests that these early developing structures incurred an insult altering their structural development. However, the structural evaluation of a single timepoint in an ongoing developmental process is a limitation. In the future, a longitudinal analysis with a second MRI evaluation is planned for this cohort as this is more sensitive to individual brain developmental patterns due to the exclusion of the influence of large interindividual variations. Also, we are unable to definitively exclude contributions from later childhood TRAP exposure with this analysis. However, given the key findings in structures that develop during the first and second trimester, it is plausible these structures have been impacted since the first year of life. It is also possible that our estimate of outdoor TRAP exposure at the participants’ homes may not reflect daily personal exposures due to home characteristics that affect the outdoor-indoor penetration of pollutants and individual time-activity patterns of participants. However, estimating health effects associated with ambient pollutant concentrations offer the potential to guide regulatory limits and public health actions on a population-level. Also, inclusion criteria for the CCAAPS cohort required having at least one biological parent with atopy. Therefore, future studies should include children born to parents with and without allergic disease to confirm the generalizability of our results.

This study design along with the SPM12 (including CAT) software may limit the sensitivity of the analysis as there may be other regions with volume or cortical thickness changes beyond our ability to detect. We acknowledge this methodology is not well-suited for assessing volume differences in structures such as the hippocampus or amygdala. To minimize imaging processing effects, we analyzed the cortical thickness data using SPM12 and also with a rival software approach, known as Freesurfer [17], where a similar result was observed, but not shown. Future studies of cohorts evaluating TRAP should also include measures of sensory-motor function.

## Conclusions

Our study found that children with higher levels of exposure to TRAP demonstrated regional reductions of cortical thickness and gray matter volume relative to children with lower levels. Reduced cortical thickness and volume loss in our study population are on the order of 3–4%. These findings are consistent with a process damaging the development of the sensorimotor cortex, frontal cortex, cerebellar vermis and cerebellar hemispheres as these structures form early in development and are vulnerable to injury.

## Supporting information

S1 Fig VBM and cortical thickness workflows in CAT12 toolbox. (TIF)
