# Supplementary figures and images for: Reduced gray matter volume and cortical thickness associated with traffic-related air pollution in a longitudinally studied pediatric cohort
Source: PLoS One. 2020 Jan 24;15(1):e0228092. doi: 10.1371/journal.pone.0228092 (PMC6980590; doi:10.1371/journal.pone.0228092)

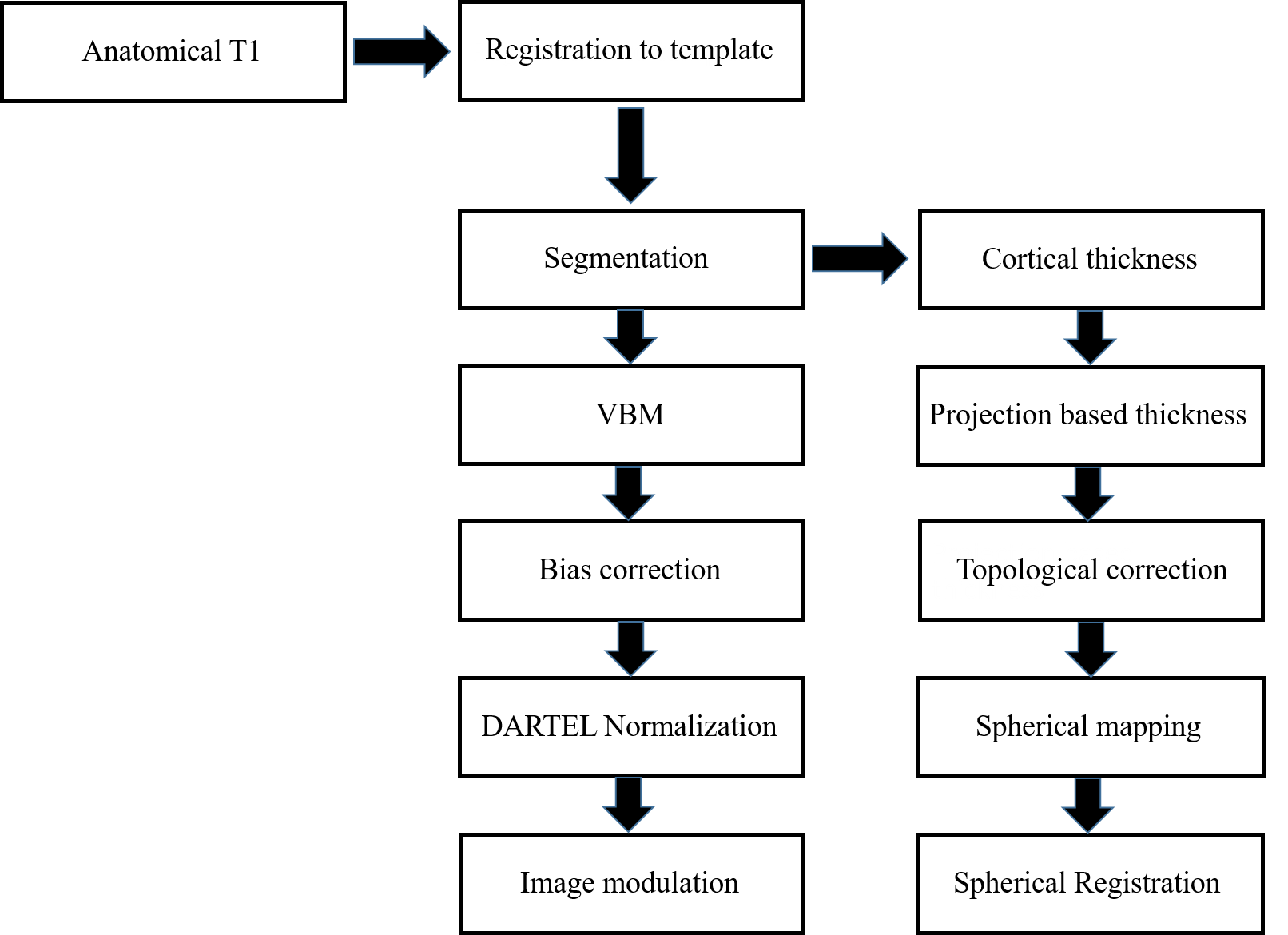

Supplement: S1 Fig — (TIF) [file pone.0228092.s001.tif]
